# The Use of Massage in the Treatment of Fractures

**Published:** 1901-08-31

**Authors:** 


### THE USE OF MASSAGE IN THE TREATMENT OF FRACTURES.

Sir William H. Bennett[1] says that a prolonged experience of the use of the combined methods of massage, early movements, and rational posture in the treatment of ordinary fractures coming under notice almost daily in hospital work, confirms the favourable estimate formed by him three years ago, and leads him to conclude that when managed with ordinary discretion and average dexterity the result of the method is undoubtedly advantageous, inasmuch as the time elapsing before the patient is able to resume his ordinary vocation is diminished by at least one-third, partly by the increased rapidity of union which ensues, and to a great extent by the avoidance of the waste of time which occurs in correcting the stiffness and pain which so often follow upon the discontinuance of splints in cases treated by the classical method of prolonged splinting, etc. The benefit of the method is remarkably demonstrated in those fractures in which the chances of bony union are practically nil—*e.g.*, intracapsular fracture of the neck of the femur—cases in which the indications are to obtain the best use in the damaged limb by ensuring free movement and by preventing the wasting of the muscles concerned. In such cases massage and passive movements are indicated at once. The method, however, is not suited to surgeons who lack discretion or who are defective in dexterity—a remark which applies with equal force to the majority of surgical procedures; to such the classical treatment by prolonged splinting, whatever its disadvantages may be, is better adapted. The method is not to be regarded as a substitute for treatment by splints on the one hand, or by operative measures on the other, but should be used as a rational adjunct to each. It is to be noted that when saying that the principal disabilities attaching to the union of fractures in faulty positions are avoidable by the use of massage and early movement, Sir William Bennett excepts cases in which the displacement is "gross or of the rotatory kind"—an exception of some importance.

[1] Practitioner, Aug.

